# Antimicrobial susceptibility to polymyxin B and other comparators against Gram-negative bacteria isolated from bloodstream infections in China: Results from CARVIS-NET program

**DOI:** 10.3389/fmicb.2022.1017488

**Published:** 2022-10-06

**Authors:** Jingyuan Xi, Peiyao Jia, Ying Zhu, Wei Yu, Jingjia Zhang, Haotian Gao, Wei Kang, Ge Zhang, Jin Li, Tong Wang, Yingchun Xu, Qiwen Yang

**Affiliations:** ^1^Department of Clinical Laboratory, State Key Laboratory of Complex Severe and Rare Diseases, Peking Union Medical College Hospital, Chinese Academy of Medical Sciences and Peking Union Medical College, Beijing, China; ^2^Department of Clinical Laboratory Center, Beijing Children’s Hospital, Capital Medical University, National Center for Children’s Health, Beijing, China; ^3^Graduate School, Peking Union Medical College, Chinese Academy of Medical Sciences, Beijing, China

**Keywords:** Gram-negative bacteria, drug resistance, bloodstream infection, resistance surveillance, polymyxin B

## Abstract

**Objective:**

To investigate the bacterial distribution and antimicrobial resistance profile of clinical isolates from Gram-negative bacteria bloodstream infections (GNBSI) in China.

**Methods:**

The clinical bacterial strains isolated from blood culture were collected during April 2019 to December 2021 in 21 member hospitals of China Bloodstream Gram-negative Pathogens Antimicrobial Resistance and Virulence Surveillance Network (CARVIS-NET). Antibiotic susceptibility test was conducted by broth microdilution method recommended by Clinical and Laboratory Standards Institute (CLSI, United States). WHONET 2021 and SPSS 22.0 were used to analyze data.

**Results:**

During the study period, 1939 Gram-negative bacteria were collected from 21 hospitals, among which 1,724 (88.9%) were *Enterobacteriaceae*, 207 (10.7%) were non-fermenting Gram-negative bacteria and 8 (0.4%) were others. The top five bacterial species were *Escherichia coli* (46.2%), *Klebsiella pneumoniae* (31.6%), *Pseudomonas aeruginosa* (4.9%), *Acinetobacter baumannii* (4.2%) and *Enterobacter cloacae* (3.0%). For *K. pneumoniae*, antibiotic resistance was mainly prevalent in hospital-associated bloodstream infections, while for *A. baumannii*, antibiotic resistance was mainly prevalent in community-associated bloodstream infections. It is worth mentioning that 94.1% of the 1939 Gram-negative isolates were susceptible to polymyxin B. The sensitivity of the strains involved in our investigation to polymyxin B is highly correlated with their sensitivity to colistin.

**Conclusion:**

The surveillance results in CARVIS-NET-2021 showed that the main pathogens of GNBSI in China were *Enterobacteriaceae*, while *E. coli* was the most common pathogen. The resistance rates of *K. pneumonia*, *P. aeruginosa*, *A. baumannii*, and *E. cloacae* to multiple antibiotics kept on a high level. In many cases, polymyxin B and colistin has become the last-resort agents to combat bloodstream infections caused by multidrug-resistant (MDR) Gram-negative bacteria.

## Introduction

Good surveillance programs are the key to combat antibiotic resistance, including preservation and transportation of strains, determination of antibiotic susceptibility rates and detection of the expression of resistance genes that spread through different populations(Critchley and Karlowsky, 2004). Many current surveillance programs have problems to be solved such as obvious duplicate clones, lacking of clinical information or incorrect identification of colonizing and infecting strains. Therefore, in order to ensure the quality of monitoring, it is necessary to designate a center laboratory for quality confirmation, and to analyze the collected data after making sure the collected pathogens are reliable (Critchley and Karlowsky, 2004; Johnson, 2015).

Gram-negative bacteria bloodstream infection (GNBSI) has been a global public health problem (Huh et al., 2020; Nutman et al., 2021). Drug resistance in Gram-negative bacteria, including those that cause bloodstream infections, has increased dramatically over the past 20 years (Ergonul et al., 2016; Huh et al., 2020). Currently, the pathogenic spectrum and the resistance characteristics of bloodstream infection in China are changing, and they are not consistent in different regions. Therefore, this topic deserves further study (Liu et al., 2018; Liang et al., 2021).

Gram-negative pathogens that produce extended-spectrum β-lactamases (ESBLs) are now common and are highly associated with nonstandard drug administration and high mortality. In addition, carbapenem resistance is also becoming increasingly severe (Rodriguez-Bano et al., 2018; Tamma et al., 2021). These objective conditions have limited clinical medication decision, so a better understanding of local epidemiology might be helpful to optimize the therapeutic regimen (Critchley and Karlowsky, 2004). It is a pity that the development of antimicrobials has not kept pace with the increasing spread of resistance genes, especially for Gram-negative bacteria. Therefore, it is critical to make better use of existing antibiotics and strict infection control scheme to prevent these life-threatening infections (Collignon and McEwen, 2019).

Polymyxins are a group of polycationic antimicrobial lipopeptides biosynthesized by bacteria belonging to the *Genus Bacillus*. Polymyxin B and polymyxin E (also known as colistin) are the major representatives of polymyxins which have been used in clinical practice since the 1950s (El-Sayed Ahmed et al., 2020). Subsequently, the emergence of significant side-effects such as neurotoxicity, nephrotoxicity and anaphylactoid reaction limited their use (Zhan et al., 2019). Nevertheless, the increase of infections caused by MDR bacteria has led to a renewed clinical emphasis on polymyxins. Polymyxins have strong antibacterial activity and are considered to be the last therapeutic agent for infections caused by a variety of multidrug-resistant Gram-negative bacteria (Nang et al., 2021).

In view of the above, we designed this study to investigate the bacterial distribution and drug resistance of clinical isolates from GNBSI in China by implementing a well-designed surveillance plan, with a view to providing factual evidence for more rational use of antibiotics in clinic.

## Materials and methods

### Bacterial strains

Gram-negative bacteria were consecutively and non-repetitively collected from patients with clinical- and laboratory-confirmed bloodstream infection between 2019 and 2021 in 21 centers located in 20 Chinese cities. All organisms were considered clinically significant by local hospital criteria and were isolated from high-quality specimens of each patient’s first positive blood culture. In this study, community-associated bloodstream infection was defined as bloodstream infection that occurred in the community (e.g., outpatient, emergency-patient blood culture-positive cases) or within 48 h of hospitalization (positive blood culture reported within 48 h of hospitalization). While nosocomial-associated bloodstream infection was defined as bloodstream infection after 48 h of hospitalization (positive blood cultures sent after 48 h of hospitalization). All isolates were sent to the central clinical microbiology laboratory of Peking Union Medical College Hospital (PUMCH) for identification confirmation by MALDI-TOF MS (Vitek MS, biomérieux, France). The Human Research Ethics Committee of PUMCH approved this study (Ethics Approval Number: HS2755).

### Antimicrobial susceptibility testing

Antimicrobial susceptibility testing was conducted by broth microdilution method as per the CLSI recommendations. MICs were interpreted following the CLSI M100-S32 guidelines (CLSI, 2022) or European Committee on Antimicrobial Susceptibility Testing (EUCAST) (2022) Breakpoint tables for interpretation of MICs and zone diameters Version 12.0. The breakpoint for *E. coli*, *K. pneumoniae* and *E. cloacae* of aztreonam/avibactam and sitafloxacin refers to the breakpoint of aztreonam and moxifloxacin, respectively. For *P. aeruginosa*, the breakpoint of aztreonam/avibactam, sitafloxacin and cefoperazone/sulbactam refers to the breakpoint of aztreonam, levofloxacin and cefoperazone, respectively. For *A. baumannii*, the breakpoint of ceftazidime/avibactam and sitafloxacin refers to the breakpoint of ceftazidime and levofloxacin, respectively. *E. coli* ATCC 25922, *P. aeruginosa* ATCC 27853 and *K. pneumoniae* ATCC 700603 were used as quality controls.

### Statistical analyses

Statistical analysis was performed with WHONET 2021 and SPSS 22.0. MIC50 and MIC90 refer to the minimum inhibitory concentration required to inhibit the growth of 50 and 90% of the tested bacteria. The 95% confidence intervals were calculated using the adjusted Wald method. Comparison of rates was assessed using a chi-squared test. Binary logistic analysis was used for independent risk factor analysis. A value of *p* < 0.05 was considered statistically significant, and *p* < 0.001 was extremely significant.

## Results

### Demographic information and pathogen distribution of patients with Gram-negative bacteria bloodstream infection

A total of 1939 Gram-negative bacteria isolated from 1939 patients with clinical- and laboratory-confirmed bloodstream infection were involved in this study. 1,102 (56.8%) were males and 837 (43.2%) were females. *Enterobacteriaceae* was the main family of pathogens isolated (1724, 88.9%), with *E. coli* (896, 46.2%) and *K. pneumoniae* (612, 31.6%) being the top two most commonly isolated species. Non-fermenting Gram-negative bacteria comprised 10.7% of the observed pathogens, of which *P. aeruginosa* (94, 4.9%) and *A. baumannii* (82, 4.2%) were the most common (Supplementary Table S1). When the infected population was divided into three groups with the boundaries of 18 and 65 years old, it was found that *E. coli* and *K. pneumoniae* were the top two Gram-negative bacteria in all the three age groups. Meanwhile, the proportion of *E. coli* appeared to increase with age (Supplementary Table S1).

### Age characteristics of patients with Gram-negative bacteria bloodstream infection

**Figure 1:**
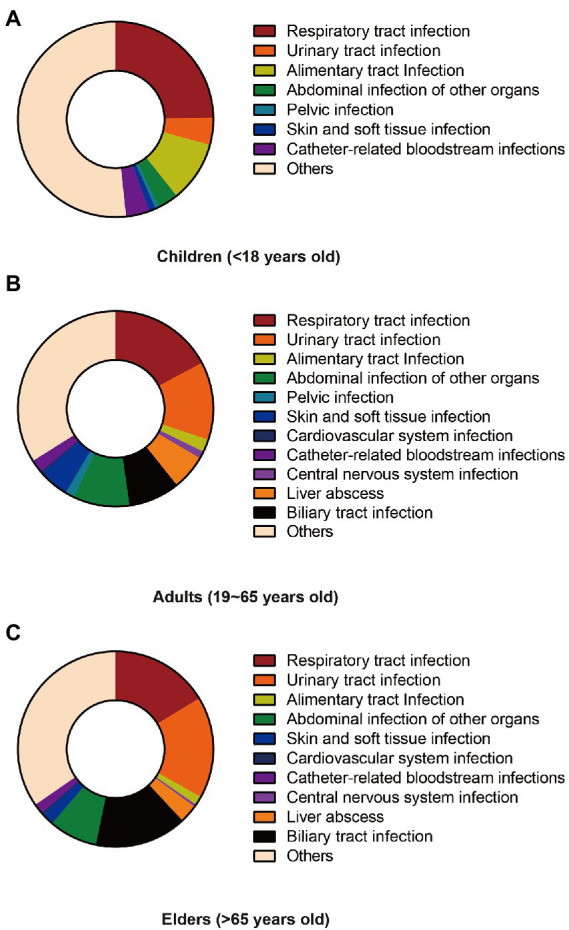
Difference infection sources of different age groups. **(A)** Composition of sources of infection among children under 18. **(B)** Composition of sources of infection among adults aged 19–65. **(C)** The composition of the source of infection in the elderly over 65 years old.

Analysis of the source of bloodstream infection showed that in the juveniles’ population, Gram-negative bacteria infection from respiratory tract contributed the most to bloodstream infection, accounting for 24.7% of all juveniles with bloodstream infection, followed by gastrointestinal infection (10.1%) and urinary tract infections (4.5%). For adults aged 19 to 65, respiratory tract infection (17.2%), urinary tract infections (12.9%) and abdominal infection of organs except for liver and gallbladder (9.5%) were the top three sources of bloodstream infections, respectively. Yet for the elderly over 65 years old, they are urinary tract infection (16.8%), respiratory tract infection (16.3%) and biliary tract infection (15.2%) in order (Figure 1). With increasing age, the proportion of primary and bloodstream Gram-negative bacteria infections that came from the community rather than the hospital increased, and so did the mortality rate (*p* < 0.001; Supplementary Table S2).

### Susceptibility of Gram-negative bacteria to antibiotics and their clinical features

#### Escherichia coli

As is shown in Supplementary Table S3, majority of the 896 strains of *E. coli* had high susceptibility to polymyxin B, colistin, ceftazidime/avibactam, aztreonam/avibactam, amikacin, meropenem, imipenem, piperacillin/tazobactam, ertapenem, cefoxitin and cefoperazone/sulbactam. Nevertheless, ceftazidime, cefepime, sitafloxacin, levofloxacin, ceftriaxone and trimethoprim/sulfamethoxazole showed poor performance in anti-*E. coli* in bloodstream infection, with resistance rates of 23.2, 32.5, 50.9, 52.1, 57.8 and 65.3%, respectively.

**Figure 2:**
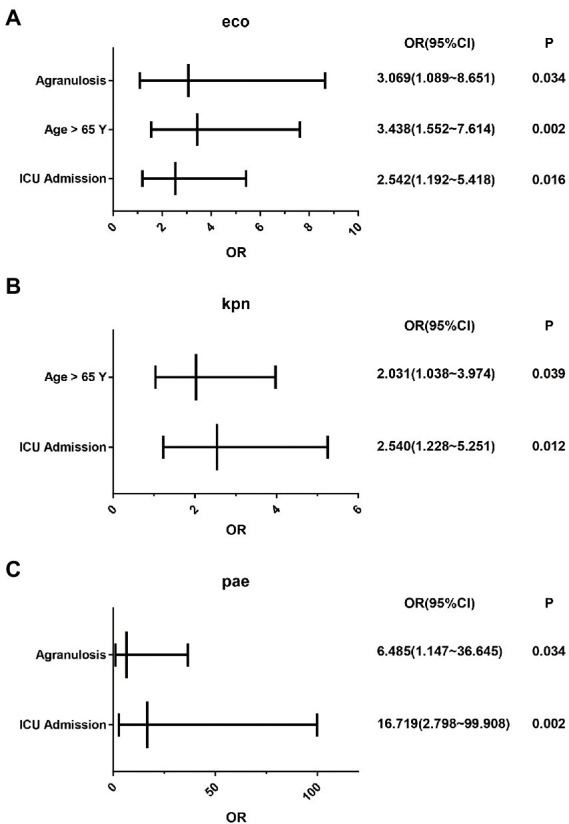
Analysis of independent risk factors for death in patients with Gram-negative bacteria bloodstream infection. **(A)** Independent risk factors for death in patients with bloodstream infection with *E. coli*. **(B)** Independent risk factors for death in patients with bloodstream infection with *K. pneumoniae*. **(C)** Independent risk factors for death in patients with bloodstream infection with *P. aeruginosa*.

Further analysis indicated that *E. coli* isolated from patients over 65 years old were more susceptible to trimethoprim/sulfamethoxazole (*p* = 0.003). Patients whose primary or bloodstream infection associated with medical institutions had lower frequency of susceptibility to trimethoprim/sulfamethoxazole (*p* < 0.01), while patients whose bloodstream infection occurred after 48 h of hospitalization had lower frequency of susceptibility to ceftazidime as well (*p* = 0.046). Among patients admitted to intensive care unit (ICU), the frequency of drug resistance to ertapenem, ceftriaxone, cefepime and ceftazidime was significantly higher than that of other patients. Curiously, patients who were more susceptible to trimethoprim/sulfamethoxazole appeared to have a higher mortality rate (*p* = 0.047; Table 1). Further binary logistic analysis found that age greater than 65 years, ICU admission, and agranulosis were independent risk factors for death in patients with *E. coli* bloodstream infection (Figure 2A).

**Table 1:** Comparison of the antimicrobial susceptibility rates of *Escherichia coli* isolates in clinical features.

Antibiotic	%S	MIC50	MIC90	Gender (%)			Age (%)			Primary infection(%)			Bloodstream infection(%)			ICU admission(%)			Clinical outcome(%)		
				Female *N* = 442	Male *N* = 454	*p*-Value	≤ 65 *N* = 506	> 65 *N* = 385	*p*-Value	CO *N* = 497	HA *N* = 348	*p*-Value	CO *N* = 504	HA *N* = 390	*p*-Value	Yes *N* = 127	No *N* = 729	*p*-Value	Death *N* = 36	Others *N* = 834	*p*-Value
Polymyxin B	99.0	0.25	0.5	98.6	99.3	0.335	98.8	99.2	0.739	99.4	98.6	0.285	99.2	98.7	0.698	98.8	99.0	1.000	97.2	99.0	0.318
Colistin	98.5	0.25	0.5	98.2	98.9	0.375	98.0	99.2	0.140	98.8	98.3	0.742	98.8	98.2	0.454	98.2	98.6	0.956	97.2	98.7	0.400
Ceftazidime/avibactam	99.8	0.064	0.064	99.8	99.8	1.000	99.8	99.7	1.000	99.8	99.7	1.000	100.0	99.5	0.190	100.0	99.7	1.000	100.0	99.8	1.000
Aztreonam/Avibactam	99.7	0.064	0.064	99.5	99.8	0.620	99.4	100.0	0.263	99.8	99.4	0.572	100.0	99.2	0.083	98.8	99.9	0.091	100.0	99.6	1.000
Amikacin	98.0	2	4	98.0	98.0	0.954	97.6	98.4	0.393	98.4	98.0	0.663	98.0	97.9	0.943	96.4	98.4	0.190	100.0	98.1	1.000
Meropenem	96.9	0.064	0.064	97.1	96.7	0.755	96.4	97.7	0.293	97.0	96.8	0.906	97.4	96.2	0.281	97.0	96.8	0.914	100.0	96.9	0.565
Imipenem	96.5	0.25	0.5	96.6	96.5	0.915	96.4	97.1	0.560	97.0	96.0	0.430	97.4	95.4	0.099	97.0	96.4	0.715	100.0	96.5	0.507
Piperacillin/Tazobactam	94.8	8	8	95.7	93.8	0.210	93.9	96.1	0.136	95.2	94.3	0.554	95.6	93.6	0.174	95.8	94.5	0.498	100.0	94.6	0.295
Ertapenem	92.7	0.064	0.25	93.9	91.6	0.192	92.1	93.8	0.338	92.8	93.1	0.847	92.9	92.6	0.867	88.0	93.8	0.009^*^	100.0	92.4	0.166
Cefoxitin	86.6	2	16	88.0	85.2	0.224	86.2	87.5	0.551	87.7	85.6	0.375	87.9	84.9	0.188	89.2	86.0	0.271	91.7	86.2	0.492
Cefoperazone/Sulbactam	85.0	8	32	86.2	83.9	0.339	83.8	86.8	0.220	85.7	83.9	0.470	85.9	83.8	0.391	80.8	86.0	0.091	86.1	85.3	0.887
Ceftazidime	69.0	1	64	71.3	66.7	0.143	67.4	71.2	0.227	71.8	66.1	0.075	71.6	65.4	0.046^*^	59.3	71.2	0.003^*^	80.6	68.3	0.121
Cefepime	53.5	2	128	54.1	52.9	0.717	52.0	55.6	0.285	55.3	52.3	0.384	55.0	51.3	0.274	44.3	55.6	0.009^*^	58.3	53.2	0.548
Sitafloxacin	49.1	0.5	2	49.3	48.9	0.899	46.8	52.2	0.112	50.3	49.7	0.866	48.8	49.5	0.841	45.5	49.9	0.302	38.9	49.5	0.212
Levofloxacin	42.6	2	16	45.0	40.3	0.154	40.1	45.7	0.094	43.9	44.0	0.976	42.1	43.3	0.703	41.3	42.9	0.703	36.1	42.8	0.426
Ceftriaxone	42.2	64	256	42.8	41.6	0.732	40.3	44.9	0.167	44.1	42.2	0.599	43.8	40.0	0.248	35.3	43.8	0.047^*^	44.4	42.0	0.768
Trimethoprim/sulfamethoxazole	34.7	8	8	34.4	35.0	0.842	30.4	40.0	0.003^*^	38.8	29.0	0.003^*^	39.3	28.7	0.001^*^	34.1	34.8	0.862	50.0	33.9	0.047^*^

* *p* < 0.05.

#### Klebsiella pneumoniae

In the cases of *K. pneumoniae*, only polymyxin B, colistin, aztreonam/avibactam and ceftazidime/avibactam had sensitivity rates greater than 80%. Conversely, amikacin, meropenem, imipenem, ertapenem, piperacillin/tazobactam, cefoperazone/sulbactam, cefoxitin, sitafloxacin, ceftazidime, levofloxacin, cefepime, ceftriaxone and trimethoprim/sulfamethoxazole showed poor performance in anti-*K. pneumoniae* bloodstream infection (Supplementary Table S4).

Further analysis indicated that *K. pneumoniae* isolated from patients over 65 years old were more susceptible to meropenem (*p* = 0.039) and imipenem (*p* = 0.022). For antibiotics except for aztreonam/avibactam, ceftazidime/avibactam, polymyxin B and colistin, isolates derived from patients whose infection occurred after 48 h of hospitalization had lower frequency of susceptibility to the antibiotics with poor performance (*p* < 0.05). Among patients admitted to ICU, the frequency of drug resistance to sitafloxacin, amikacin, ertapenem, meropenem, piperacillin/tazobactam, cefepime, cefoperazone/sulbactam, ceftriaxone, ceftazidime, cefoxitin, imipenem and levofloxacin was significantly higher than that of other patients (*p* < 0.05). The mortality of these patients was higher than others as well (*p* < 0.001; Table 2). Binary logistic analysis found that age greater than 65 years and ICU admission were independent risk factors for death in patients with *K. pneumoniae* bloodstream infection (Figure 2B).

**Table 2:** Comparison of the antimicrobial susceptibility rates of *K. pneumoniae* isolates in clinical features.

Antibiotic	%S	MIC50	MIC90	Gender (%)			Age (%)			Primary infection(%)			Bloodstream infection(%)			ICU admission(%)			Clinical outcome(%)		
				Female *N* = 219	Male *N* = 393	*p*-value	≤ 65 *N* = 401	> 65 *N* = 200	*p*-Value	CO *N* = 308	HA *N* = 280	*p*-Value	CO *N* = 292	HA *N* = 317	*p*-Value	Yes *N* = 196	No *N* = 416	*p*-Value	Death *N* = 55	Others *N* = 528	*p*-Value
Polymyxin B	97.1	0.5	0.5	96.3	97.5	0.437	97.3	96.4	0.575	97.7	97.9	0.915	96.9	97.2	0.860	96.9	97.1	0.904	94.5	97.2	0.236
Colistin	97.1	0.25	0.5	96.3	97.5	0.437	97.3	96.4	0.575	97.7	97.9	0.915	96.9	97.2	0.860	96.9	97.1	0.904	94.5	97.2	0.236
Aztreonam/Avibactam	99.8	0.064	0.064	100.0	99.7	1.000	100.0	99.5	0.722	100.0	99.6	0.962	100.0	99.7	1.000	99.5	100.0	0.700	98.2	100.0	0.165
Ceftazidime/avibactam	99.5	0.064	0.064	100.0	99.2	0.489	99.5	99.5	1.000	100.0	98.9	0.214	100.0	99.1	0.277	100.0	99.3	0.568	100.0	99.4	1.000
Amikacin	78.3	1	128	78.1	78.4	0.934	77.5	80.2	0.446	86.0	71.1	0.000^*^	87.7	69.4	0.000^*^	66.3	83.9	0.000^*^	47.3	81.1	0.000^*^
Meropenem	74.8	0.064	256	78.1	73.3	0.189	73.0	80.7	0.039^*^	86.0	64.3	0.000^*^	87.7	62.8	0.000^*^	60.7	81.5	0.000^*^	49.1	76.7	0.000^*^
Imipenem	73.7	0.25	128	76.7	72.0	0.205	71.5	80.2	0.022^*^	84.1	63.9	0.000^*^	85.6	62.5	0.000^*^	61.2	79.6	0.000^*^	49.1	75.6	0.000^*^
Ertapenem	71.4	0.064	256	74.0	70.0	0.294	70.0	76.1	0.118	82.1	61.1	0.000^*^	83.9	59.6	0.000^*^	57.7	77.9	0.000^*^	45.5	73.3	0.000^*^
Piperacillin/Tazobactam	71.4	8	> 256	74.9	69.5	0.155	70.0	76.6	0.090	80.8	62.5	0.000^*^	83.6	59.9	0.000^*^	57.7	77.9	0.000^*^	47.3	73.5	0.000^*^
Cefoperazone/Sulbactam	67.2	8	256	65.3	68.2	0.465	66.6	70.1	0.394	77.9	56.8	0.000^*^	80.1	54.9	0.000^*^	51.5	74.5	0.000^*^	36.4	69.3	0.000^*^
Cefoxitin	64.7	2	128	64.8	64.6	0.959	63.6	69.0	0.190	75.6	55.4	0.000^*^	76.7	53.9	0.000^*^	53.1	70.2	0.000^*^	34.5	67.2	0.000^*^
Sitafloxacin	63.7	0.125	16	65.3	62.8	0.546	63.6	65.0	0.744	72.1	56.1	0.000^*^	73.6	54.3	0.000^*^	51.5	69.5	0.000^*^	32.7	66.1	0.000^*^
Ceftazidime	63.1	0.5	256	64.8	62.1	0.499	61.4	68.0	0.113	73.4	53.9	0.000^*^	76.0	51.4	0.000^*^	53.6	67.5	0.001^*^	32.7	65.5	0.000^*^
Levofloxacin	58.7	0.5	64	58.4	58.8	0.936	58.4	59.9	0.729	66.6	51.8	0.000^*^	67.8	49.8	0.000^*^	45.4	64.9	0.000^*^	25.5	61.2	0.000^*^
Cefepime	57.0	0.125	256	53.4	59.0	0.179	55.9	60.4	0.299	69.2	45.0	0.000^*^	71.6	43.2	0.000^*^	45.9	62.3	0.000^*^	32.7	58.5	0.000^*^
Ceftriaxone	52.9	0.25	256	48.4	55.5	0.093	52.0	55.8	0.374	66.6	39.6	0.000^*^	68.8	38.5	0.000^*^	43.9	57.2	0.002^*^	25.5	54.7	0.000^*^
Trimethoprim/sulfamethoxazole	52.0	2	8	48.4	53.9	0.188	52.2	51.3	0.825	60.1	42.5	0.000^*^	62.0	42.3	0.000^*^	49.0	53.4	0.311	40.0	52.3	0.083

* *p* < 0.05.

#### Pseudomonas aeruginosa

Toward *P. aeruginosa*, polymyxin B, colistin, aztreonam/avibactam, ceftazidime/avibactam, sitafloxacin and amikacin performed well. All of the other antibiotics involved in this investigation had poor sensitivities, among which there were naturally resistant ones (Supplementary Table S5). Patients primary infected in community had significantly higher frequency of susceptibility to Sitafloxacin (*p* = 0.022). Among patients admitted to ICU, the frequency of drug resistance to Levofloxacin was significantly higher than that of other patients (*p* = 0.024; Table 3). Binary logistic analysis found that agranulosis and ICU admission were independent risk factors for death in patients with *P. aeruginosa* bloodstream infection (Figure 2C).

**Table 3:** Comparison of the antimicrobial susceptibility rates of *P. aeruginosa* isolates in clinical features.

Antibiotic	%S	MIC50	MIC90	Gender (%)			Age (%)			Primary infection(%)			Bloodstream infection(%)			ICU admission(%)			Clinical outcome(%)		
				Female *N* = 31	Male *N* = 64	*p*-Value	≤ 65 *N* = 66	> 65 *N* = 28	*p*-Value	CO *N* = 34	HA *N* = 56	*p*-Value	CO *N* = 33	HA *N* = 62	*p*-Value	Yes *N* = 32	No *N* = 63	*p*-Value	Death *N* = 13	Others *N* = 82	*p*-Value
Polymyxin B	98.9	1	1	100.0	98.4	1.000	98.5	100.0	1.000	100.0	98.2	1.000	100.0	98.4	1.000	100.0	98.4	1.000	100.0	98.8	1.000
Colistin	98.9	1	1	100.0	98.4	1.000	98.5	100.0	1.000	100.0	98.2	1.000	100.0	98.4	1.000	100.0	98.4	1.000	100.0	98.8	1.000
Ceftazidime/avibactam	96.8	1	4	100.0	95.3	0.548	95.5	100.0	0.552	97.1	98.2	1.000	97.0	96.8	1.000	100.0	95.2	0.526	100.0	96.3	1.000
Amikacin	94.7	2	8	96.8	93.8	0.897	95.5	92.9	0.991	100.0	92.9	0.286	100.0	91.9	0.233	93.8	95.2	1.000	92.3	95.1	0.529
Sitafloxacin	90.5	0.125	1	93.5	89.1	0.744	92.4	85.7	0.530	100.0	85.7	0.022^*^	100.0	85.5	0.053	87.5	92.1	0.728	92.3	90.2	1.000
Aztreonam/Avibactam	85.3	4	16	83.9	85.9	1.000	84.8	85.7	1.000	94.1	83.9	0.272	90.9	82.3	0.407	81.3	87.3	0.631	76.9	86.6	0.623
Cefepime	81.1	2	32	77.4	82.8	0.529	80.3	82.1	0.836	79.4	83.9	0.587	75.8	83.9	0.337	84.4	79.4	0.556	92.3	79.3	0.463
Ceftazidime	81.1	2	64	77.4	82.8	0.529	80.3	82.1	0.836	79.4	82.1	0.748	78.8	82.3	0.681	81.3	81.0	0.972	100.0	78.0	0.135
Piperacillin/Tazobactam	76.8	8	128	77.4	76.6	0.926	80.3	67.9	0.192	76.5	76.8	0.973	78.8	75.8	0.743	78.1	76.2	0.833	92.3	74.4	0.285
Meropenem	74.7	0.5	64	77.4	73.4	0.675	75.8	71.4	0.660	88.2	71.4	0.063	84.8	69.4	0.098	75.0	74.6	0.966	69.2	75.6	0.882
Levofloxacin	73.7	0.5	8	71.0	75.0	0.676	78.8	64.3	0.140	82.4	71.4	0.242	78.8	71.0	0.410	59.4	81.0	0.024^*^	61.5	75.6	0.465
Imipenem	67.4	2	32	71.0	65.6	0.603	68.2	64.3	0.713	73.5	67.9	0.569	69.7	66.1	0.724	65.6	68.3	0.796	69.2	67.1	1.000

* *p* < 0.05.

#### Acinetobacter baumannii

For *A. baumannii*, only polymyxin B and colistin had susceptibility greater than 90%, whose susceptibility were both 96.3%. Ceftazidime/avibactam and sitafloxacin performed fairly well with susceptibility of 84.1 and 85.4%, respectively. Except for antibiotics which had no breakpoints to be referred yet, the drug resistance rate of all other antibiotics reached 60.0% and above (Supplementary Table S6). Different from *E. coli* and *K. pneumoniae*, patients infected with *A. baumannii* in the community had higher frequency of resistance to cefepime, imipenem and levofloxacin. For ICU patients, the frequency of drug resistance to amikacin, trimethoprim/sulfamethoxazole, meropenem, piperacillin/tazobactam, cefepime, ceftriaxone and levofloxacin was significantly higher than that of other patients (*p* < 0.05). Patients infected with strains resistant to trimethoprim/sulfamethoxazole and amikacin had higher mortality (*p* < 0.05; Table 4).

**Table 4:** Comparison of the antimicrobial susceptibility rates of *A. baumannii* isolates in clinical features.

Antibiotic	%S	MIC50	MIC90	Gender (%)			Age (%)			Primary infection(%)			Bloodstream infection(%)			ICU admission(%)			Clinical outcome(%)		
				Female *N* = 42	Male *N* = 40	*p*-Value	≤ 65 *N* = 59	> 65 *N* = 23	*p*-Value	CO *N* = 29	HA *N* = 53	*p*-Value	CO *N* = 20	HA *N* = 62	*p*-Value	Yes *N* = 45	No *N* = 37	*p*-Value	Death *N* = 19	Others *N* = 62	*p*-Value
Polymyxin B	96.3	0.5	1	100.0	92.5	0.112	96.6	95.7	1.000	96.6	96.2	1.000	100.0	95.2	1.000	95.6	97.3	1.000	94.7	96.8	0.557
Colistin	96.3	0.5	1	100.0	92.5	0.112	96.6	95.7	1.000	96.6	96.2	1.000	100.0	95.2	1.000	95.6	97.3	1.000	94.7	96.8	0.557
Sitafloxacin	85.4	1	4	85.7	85.0	0.927	84.7	87.0	1.000	75.9	90.6	0.140	80.0	87.1	0.677	82.2	89.2	0.374	94.7	82.3	0.332
Ceftazidime/avibactam	84.1	4	16	81.0	87.5	0.417	83.1	87.0	0.922	79.3	86.8	0.568	75.0	87.1	0.349	86.7	81.1	0.491	89.5	82.3	0.695
Amikacin	35.4	128	128	31.0	40.0	0.392	37.3	30.4	0.560	27.6	39.6	0.276	30.0	37.1	0.564	24.4	48.6	0.023^*^	15.8	40.3	0.049^*^
Trimethoprim/sulfamethoxazole	28.0	8	8	26.2	30.0	0.701	32.2	17.4	0.180	13.8	35.8	0.034	15.0	32.3	0.135	17.8	40.5	0.022^*^	5.3	35.5	0.011^*^
Imipenem	28.0	64	128	28.6	27.5	0.914	32.2	17.4	0.180	13.8	35.8	0.034^*^	15.0	32.3	0.135	20.0	37.8	0.074	15.8	32.3	0.164
Ceftazidime	25.6	64	256	21.4	30.0	0.374	28.8	17.4	0.287	13.8	32.1	0.070	20.0	27.4	0.509	17.8	35.1	0.073	15.8	29.0	0.394
Meropenem	24.4	64	128	23.8	25.0	0.900	27.1	17.4	0.357	13.8	30.2	0.098	15.0	27.4	0.409	13.3	37.8	0.010^*^	5.3	30.6	0.052
Piperacillin/Tazobactam	23.2	256	>256	23.8	22.5	0.888	27.1	13.0	0.175	13.8	28.3	0.137	15.0	25.8	0.489	13.3	35.1	0.020^*^	5.3	29.0	0.067
Levofloxacin	23.2	8	32	21.4	25.0	0.702	25.4	17.4	0.439	10.3	30.2	0.042^*^	15.0	25.8	0.489	11.1	37.8	0.004^*^	10.5	27.4	0.226
Cefepime	19.5	64	256	20.0	19.0	0.913	22.0	13.0	0.540	6.9	26.4	0.033^*^	10.0	22.6	0.363	8.9	32.4	0.007^*^	5.3	24.2	0.138
Ceftriaxone	13.4	256	256	15.0	11.9	0.681	13.6	13.0	1.000	3.4	18.9	0.105	5.0	16.1	0.372	4.4	24.3	0.021^*^	5.3	16.1	0.408
Ertapenem	1.2	256	256	0.0	2.5	0.488	0.0	4.3	0.280	0.0	1.9	1.000	0.0	1.6	1.000	0.0	2.7	0.451	0.0	1.6	1.000

* *p* < 0.05.

#### Enterobacter cloacae

*Enterobacter cloacae* isolated in this study were highly susceptible to ceftazidime/avibactam, aztreonam/avibactam, amikacin and meropenem, while their susceptibility to ertapenem, piperacillin/tazobactam and cefoperazone/sulbactam also exceeded 80%. However, for other antibiotics involved in this study, the drug resistance of *E. cloacae* in bloodstream infection could not be underestimated (Supplementary Table S7). It was found that patients over 65 years old were more susceptible to trimethoprim/sulfamethoxazole (*p* = 0.035) and cefepime (*p* = 0.036; Table 5). Notably, patients who were resistant to cefoperazone/sulbactam had a higher mortality rate (*p* = 0.027; Table 5).

**Table 5:** Comparison of the antimicrobial susceptibility rates of *E. cloacae* isolates in clinical features.

Antibiotic	%S	MIC50	MIC90	Gender (%)			Age (%)			Primary infection(%)			Bloodstream infection(%)			ICU admission(%)			Clinical outcome(%)		
				Female *N* = 20	Male *N* = 38	*p*-Value	≤ 65 *N* = 37	> 65 *N* = 21	*p*-Value	CO *N* = 18	HA *N* = 38	*p*-Value	CO *N* = 16	HA *N* = 42	*p*-Value	Yes *N* = 36	No *N* = 22	*p*-Value	Death *N* = 5	Others *N* = 50	*p*-Value
Polymyxin B	81.0	0.5	128	70.0	86.8	0.229	81.1	81.0	1.000	72.2	86.8	0.337	75.0	83.3	0.727	77.3	83.3	0.821	80.0	80.0	1.000
Colistin	79.3	0.25	128	70.0	84.2	0.353	81.1	76.2	0.917	70.2	84.2	0.487	75.0	81.0	0.891	72.7	83.3	0.526	60.0	80.0	0.298
Ceftazidime/avibactam	100.0	0.064	0.064	100.0	100.0	-	100.0	100.0	-	100.0	100.0	-	100.0	100.0	-	100.0	100.0	-	100.0	100.0	-
Aztreonam/Avibactam	98.3	0.064	0.125	100.0	97.4	1.000	97.3	100.0	1.000	100.0	100.0	-	100.0	97.6	1.000	100.0	97.2	1.000	100.0	98.0	1.000
Amikacin	96.6	1	4	95.0	97.4	1.000	94.6	100.0	0.530	94.4	100.0	0.321	93.8	97.6	0.479	100.0	94.4	0.521	100.0	96.0	1.000
Meropenem	91.4	0.064	0.25	85.0	94.7	0.328	86.5	100.0	0.148	88.9	94.7	0.587	87.5	92.9	0.609	90.9	91.7	1.000	100.0	90.0	1.000
Ertapenem	84.5	0.125	2	80.0	86.8	0.762	78.4	95.2	0.184	83.3	86.8	1.000	81.3	85.7	0.989	86.4	83.3	1.000	100.0	82.0	0.578
Piperacillin/Tazobactam	82.8	8	32	80.0	84.2	0.970	78.4	90.5	0.418	77.8	86.8	0.636	75.0	85.7	0.564	81.8	83.3	1.000	60.0	86.0	0.184
Cefoperazone/Sulbactam	82.8	8	32	80.0	84.2	0.970	78.4	90.5	0.418	77.8	84.2	0.831	75.0	85.7	0.564	72.7	88.9	0.221	40.0	88.0	0.027^*^
Imipenem	79.3	0.5	4	65.0	86.8	0.107	75.7	85.7	0.569	72.2	84.2	0.487	75.0	81.0	0.891	81.8	77.8	0.972	100.0	78.0	0.571
Sitafloxacin	77.6	0.012	2	80.0	76.3	1.000	78.4	76.2	1.000	72.2	81.6	0.654	75.0	78.6	1.000	77.3	77.8	1.000	80.0	76.0	1.000
Cefepime	77.6	0.064	128	70.0	81.6	0.500	67.6	95.2	0.036^*^	72.2	81.6	0.654	68.8	81.0	0.520	77.3	77.8	1.000	100.0	74.0	0.324
Levofloxacin	72.4	0.064	8	70.0	73.7	0.765	70.3	76.2	0.628	61.1	78.9	0.278	68.8	73.8	0.955	77.3	69.4	0.517	80.0	70.0	1.000
Ceftazidime	65.5	0.5	128	60.0	68.4	0.521	59.5	76.2	0.198	61.1	68.4	0.589	56.3	69.0	0.359	59.1	69.4	0.421	40.0	68.0	0.327
Ceftriaxone	51.7	1	256	55.0	50.0	0.717	43.2	66.7	0.086	55.6	50.0	0.698	50.0	52.4	0.871	40.9	58.3	0.198	20.0	56.0	0.178
Trimethoprim/sulfamethoxazole	48.3	4	8	45.0	50.0	0.717	37.8	66.7	0.035^*^	55.6	44.7	0.449	56.3	45.2	0.453	59.1	41.7	0.198	60.0	46.0	0.659
Cefoxitin	10.3	128	128	15.0	7.9	0.405	10.8	9.5	1.000	5.6	13.2	0.652	0.0	14.3	0.173	4.5	13.9	0.392	0.0	12.0	1.000

* *p* < 0.05.

### Correlation between susceptibility of polymyxin B/colistin and clinical background of patients

**Figure 3:**
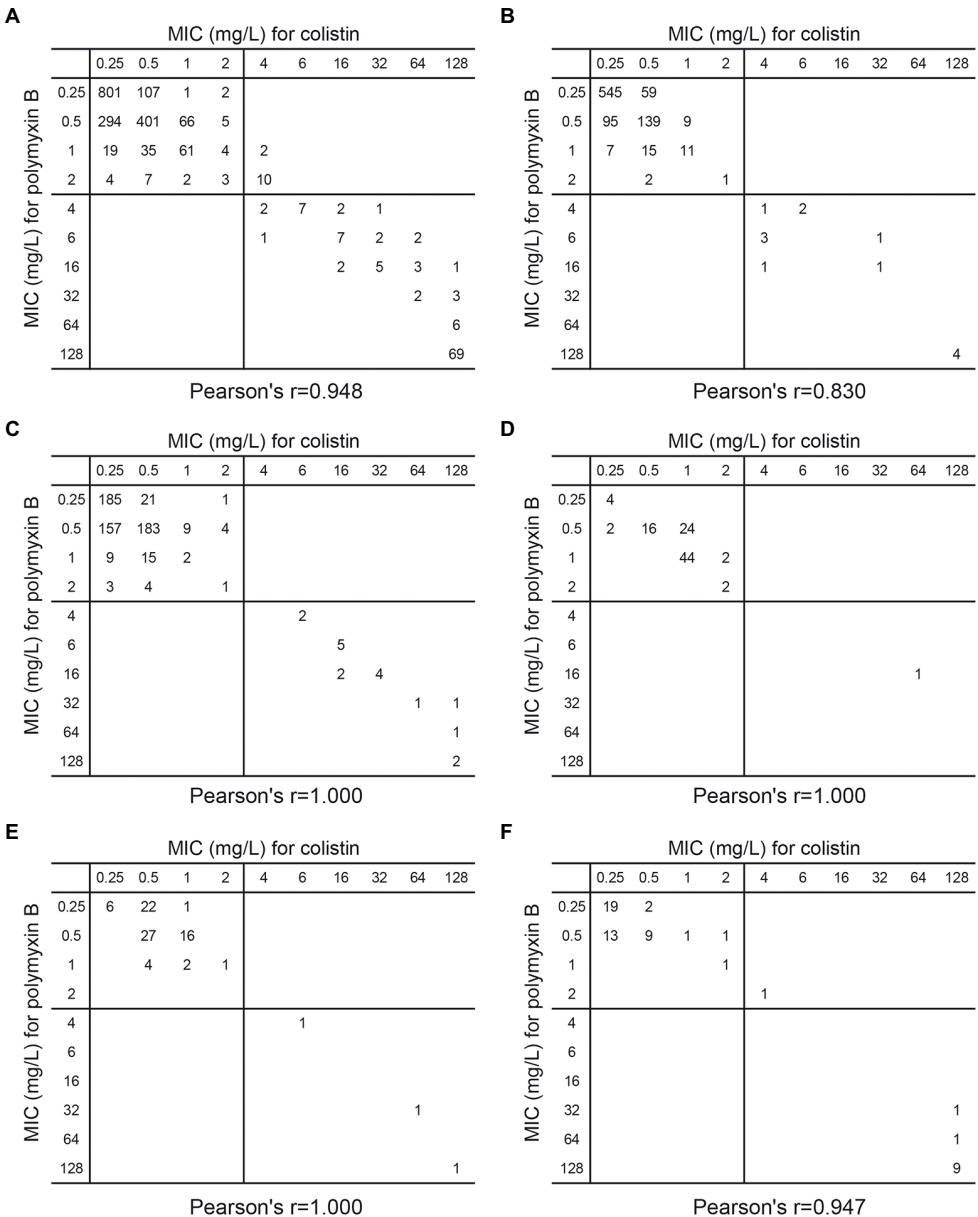
Scatterplot of MIC (mg/l) of colistin versus polymyxin B against **(A)** 1,939 Gram-negative bacteria **(B)** 896 *E. coli* **(C)** 612 *K. pneumoniae* **(D)** 95 *P. aeruginosa* **(E)** 82 *A. baumannii* **(F)** 58 *E. cloacae* which caused bloodstream infection in China.

Since both polymyxin B and colistin belonged to polypeptide antibiotics, it was wondered whether there was a correlation between the susceptibility of different pathogens towards polymyxin B and colistin. Scatterplot analyses and Fisher’s exact test revealed that when polymyxin B and colistin were used on Gram-negative bacteria causing bloodstream infection, their minimum inhibitory concentrations(mics) were significantly correlated (Pearson’s r = 0.948, *p* < 0.001). This conclusion was also valid when *E. coli* (Pearson’s r = 0.830; *p* < 0.001), *K.* (Pearson’s r = 1.000, *p* < 0.001), *P. aeruginosa* (Pearson’s *r* = 1.000; *p* < 0.001), *A. baumannii* (Pearson’s *r* = 1.000; *p* < 0.001) and *E. cloacae* (Pearson’s *r* = 0.947; *p* < 0.001) were analyzed separately (Figure 3).

Comparing the clinical background of different isolates, it was found that the susceptibility of these Gram-negative bacteria to polymyxin B and colistin did not vary significantly depending on whether the patients were suffering from pulmonary diseases, hepatobiliary diseases, digestive system diseases, cardiovascular system diseases, nervous system diseases, urinary system diseases, diabetes, agranulosis or any form of tumors. The smoking or splenectomy history did not affect the response of Gram-negative pathogens to polymyxin B or colistin either. Strangely, among strains involved in this study, which were isolated from patients with hypoproteinemia seemed to be more susceptible to polymyxin B, while those isolated from patients with history of alcohol intake were more susceptible to colistin (Supplementary Table S8). These associations needed to be further analyzed and confirmed. When it comes to recent medical operation, it was revealed that most common medical procedures occurred in recent 3 months did not seem to affect the efficacy of polymyxin B and colistin, including medical institution admission, surgery, hormone usage, immunosuppressor usage, indwelling catheter usage and antibiotics usage history. Similarly, whether the patient’s fever exceeds 39°C at this visit has no effect yet (Supplementary Table S8).

When it comes to the issue of the difference in susceptibility of Gram-negative bacteria from different infection sources to polymyxin B and colistin, it was implied that the bloodstream infection isolates from catheter-related bloodstream infections had the lowest susceptibility to polymyxin B (90.4%; Supplementary Table S9). And isolates from abdominal infection of other organs except for liver and biliary tract infection had the lowest susceptibility to colistin (90.0%; Supplementary Table S9). For alimentary tract infection and cardiovascular system infection derived Gram-negative bacterial bloodstream infection, the susceptibility of polymyxin B reached 100.0% (Supplementary Table S9).

The general geographical differences in polymyxin B and colistin susceptibilities were presented in Supplementary Table S10; Supplementary Figure S1. It was shown that Gram-negative strains from East China had the highest resistance rate to polymyxin B and colistin, which were 9.3 and 10.1% respectively, while in South China, their resistance rates were both 3.1%. To our relief, Gram-negative bacteria prevalent in most regions of China were still highly susceptible to polymyxin B (3.1% ~ 9.3%) and colistin (3.1% ~ 10.1%). The results of deep analysis showed that for *E. coli*, *K. pneumoniae*, *P. aeruginosa*, and *A. baumannii*, the susceptibility of polymyxin B in different regions of China were between 92.3 and 100.0%. While for *E. cloacae*, its sensitivities in North, East, Central, South and Northeast China were 87.5, 82.4, 71.4, 33.3 and 75.0%, separately (Supplementary Table S11).

## Discussion

According to the data collected by CARVIS-NET, in China, the pathogenic spectrum and infection source distribution of Gram-negative bacteria bloodstream infection patients of different age groups had different characteristics. And different age groups responded differently to different antibiotics. Levofloxacin has been used in China as early as 1990s, which might be the reason why the susceptibility rate of adult and elderly patients to it and its similar antibiotic amikacin was lower. Similarly, for several newer antibiotics, the susceptibility rate in the minor patient group was relatively low. Perhaps due to the poor immune function of the elderly, the proportion of patients with primary and bloodstream infections from the community and the mortality rate gradually increased with age. Whether there is a deeper reason for this remains to be further investigated.

From the drug resistance monitoring data, it could be seen that drug resistance of Gram-negative bacteria to antibiotics is still a serious problem in China. For the 2nd generation cephalosporins represented by cefoxitin, only *E. coli* had a relative low resistance rate of 9.1%, and all the other species had a resistance rate higher than 30%, or MIC50 reached 128 μg/ml. For the 3rd generation cephalosporins represented by ceftriaxone and ceftazidime, among the five common gram-negative bacteria involved in in this study, except for *P. aeruginosa*, whose resistance rate to ceftazidime was 13.7%, the resistance rates of the other four bacteria were all higher than 20%. Similarly, for cefepime, a 4th generation cephalosporin, the resistance rates of *E. coli*, *K. pneumoniae* and *A. baumannii* all exceeded 30%. It should be emphasized that the drug resistance rates of *A. baumannii* to almost all the cephalosporins involved were more than 74.0%. Based on these facts, it could be inferred that the effective rates of monotherapy with cephalosporins to treat gram-negative bacterial bloodstream infection was not satisfactory. Further investigation on susceptibility of ceftazidime combined with avibactam, a novel β-lactamase inhibitor, pointed that this combination could significantly improve the efficiency of ceftazidime. Resistance to ceftazidime for 99.1% of *E. coli*, 98.5% of *K. pneumoniae*, 100.0% of *A. baumannii*, and 100.0% of *E. cloacae* were offset by the use of Avibactam. Yet for 16.9% of ceftazidime-resistant *P. aeruginosa* strains, drug resistance was still not improved. Therefore, the presence of β-lactamase was still a great challenge for the usage of β-lactam antibiotics.

Early studies had found that many bacteria could not directly use the folic acid from environment, which was necessary for the synthesis of purine and pyrimidine(Gleckman et al., 1981). Trimethoprim/sulfamethoxazole(SXT) was designed to target on the first two steps of folic acid anabolism, respectively, so that the synthesis of folic acid could be fundamentally blocked(Smilack, 1999). At first, the effect of SXT was satisfactory. However, with its rapid promotion clinically, Gram-negative bacteria had gradually optimized their metabolic pathways under the strong evolutionary pressure. Therefore, the current situation of resistance to sulfonamides was also not optimistic.

For Gram-negative bacteria, quinolones mainly target bacterial DNA gyrase(Hooper, 1998; Hooper and Jacoby, 2015). The application of quinolones began in the 1980s. With the extensive application in the past 40 years, the response rate of quinolones in Gram-negative bacteria was getting lower and lower, and the problem of drug resistance could not be ignored either.

In addition to the antibiotics with common drug resistance problem above, different bacteria have their own characteristics in response to other antibiotics. For *E. coli*, glycylcyclones/halotetracyclines, carbapenems, penicillins or cephalosporins combined with β-lactamase inhibitors performed well. For *K. pneumoniae*, whose problem of drug resistance was getting increasingly serious, β-lactams or the third-generation cephalosporins acted only when β-lactamase inhibitor avibactam was combined. *P. aeruginosa* was naturally resistant to ertapenem and ceftriaxone involved in this investigation. When ceftazidime was combined with avibactam, its inhibitory effect was also remarkable. When it comes to *E. cloacae*, the sensitivity rate when ceftazidime was used for single drug treatment was only 65.5%, yet when combined with avibactam, the sensitivity rate was greatly improved to 100%. The underlying mechanism was that in recent years, the prevalence of ESBL among Gram-negative bacteria has shown an upward trend, and many bacteria have acquired β-lactamases, which could lead β-lactam antibiotics (including ceftazidime) to be hydrolyzed, thus losing the inhibitory effect on the strain. Avibactam belongs to β-lactamase inhibitor. When ceftazidime and avibactam are applied simultaneously, the β-lactamases will be inhibited so that they no longer have the ability to degrade ceftazidime, thus they are still sensitive to ceftazidime (Castanheira et al., 2021; Yahav et al., 2021). Besides, amikacin, an aminoglycoside, performed well against *P. aeruginosa* and *E. cloacae* in bloodstream infections as well.

Polymyxins were important antibiotics for the treatment of Gram-negative bacteria, especially MDR Gram-negative bacteria(Nang et al., 2021). In the past, colistin have been widely used in animal husbandry. The long-term irregular application has led to the gradual development of resistance of gram-negative bacteria from animals to colistin. In 2015, MCR-1, the first plasmid mediated colistin resistance gene in enterobacteriaceae in china, was detected. Some researchers found that the detection rates of MCR-1 in samples of raw meat, animals and infected inpatients were 15, 21 and 1%, respectively. The difference was because that colistin have large side effects on humans, especially for kidney, so their use in humans was limited. Recently, due to the increase of infections caused by multidrug-resistant Gram-negative bacteria, colistin and polymyxin have been reapplied globally as “the last antibiotic,” but it is far less than its early application in animal husbandry in terms of frequency and dosage. Therefore, although the resistance rate of bacteria from animals is high at present, it still maintains high sensitivity rate in humans (Pitt et al., 2003; Landman et al., 2005; Keen 3rd et al., 2010; Gales et al., 2011; Quan et al., 2017). However, its high drug resistance in animal husbandry was a potentially hidden danger thence monitoring the susceptibility of them also had important academic and clinical value (Rhouma et al., 2016). It is worth emphasizing that in this research, polymyxins represented by polymyxin B and colistin had outstanding performance against Gram-negative bacteria represented by *E. coli*, *K. pneumoniae*, *P. aeruginosa* and *A. baumannii*. When used to resist the bloodstream infection of these four bacteria, the susceptibility rates reached 95% unexceptionally. In this cohort, polymyxin B showed relatively poor performance in anti-*E. cloacae*. Considering the limited sample size, large sample studies were still necessary.

In conclusion, the surveillance results in CARVIS-NET showed that the main pathogens of Gram-negative bacteria bloodstream infection in China were *Enterobacteriaceae*, while *E. coli* accounted for the vast majority. The resistance rate of *K. pneumoniae*, *P. aeruginosa*, *A. baumannii* and *E. cloacae* to multiple antibiotics kept on a high level. In many cases, polymyxin B and colistin has become the last-resort agents to combat bloodstream infections caused by MDR Gram-negative bacteria.

## Data availability statement

The original contributions presented in the study are included in the article/supplementary material, further inquiries can be directed to the corresponding author.

## Author contributions

QY conceived and designed the study. JX analyzed the data and wrote the manuscript. PJ, YZ, WY, QY, and YX revised the manuscript. JX, JZ, HG, WK, GZ, JL, and TW performed the experiments. All authors contributed to the article and approved the submitted version.

## Funding

This work was supported by the National Natural Science Foundation of China (82072318) and National Key Research and Development Program of China (2021YFC2301002). This work was also supported by Wuhan Healcare Pharmaceuticals Co., Ltd. The funders had no role in study design, data collection and analysis, decision to publish or preparation of the manuscript. We are grateful to all the investigators of this study.

## Conflict of interest

The authors declare that the research was conducted in the absence of any commercial or financial relationships that could be construed as a potential conflict of interest.

## Publisher’s note

All claims expressed in this article are solely those of the authors and do not necessarily represent those of their affiliated organizations, or those of the publisher, the editors and the reviewers. Any product that may be evaluated in this article, or claim that may be made by its manufacturer, is not guaranteed or endorsed by the publisher.
