# Supplementary material for: Antimicrobial susceptibility to polymyxin B and other comparators against Gram-negative bacteria isolated from bloodstream infections in China: Results from CARVIS-NET program
Source: Front Microbiol. 2022 Oct 6;13:1017488. doi: 10.3389/fmicb.2022.1017488 (PMC9582771; doi:10.3389/fmicb.2022.1017488)
Supplement: Supplementary file 1 [file Data_Sheet_1.PDF]

Supplementary Material

Supplementary Figure 1. Geographical distribution of the 21 sub-centers involved in this investigation (Each black dot represents for a sub-center.). The 21 sub-centers involved in this study are located in Harbin, Shenyang, Beijing, Shijiazhuang, Taiyuan, Xi'an, Urumqi, Zhengzhou, Wuhan, Changsha, Nanchang, Chongqing, Chengdu, Kunming, Shanghai, Jinan, Hefei, Hangzhou, Fuzhou and Guangzhou, respectively.

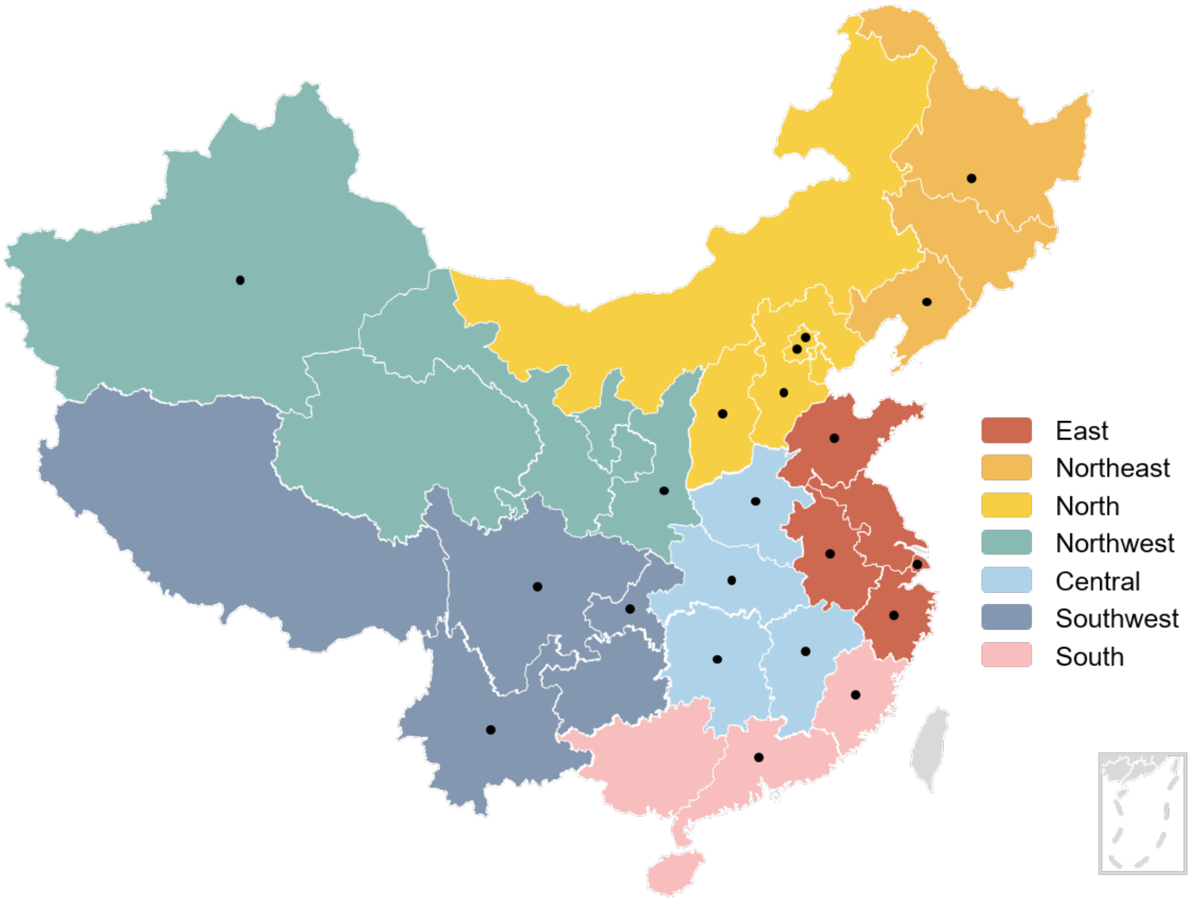

Supplementary Table 1. Distribution and age characteristics of gram-negative bacteria which caused bloodstream infection in 21 hospitals of China.

Pathogen distribution [N (percentage)]*	Total	≤18 years old	19~65 years old	＞65 years old
**Enterobacteriaceae**				
E. coli	896(46.2)	55(34.2)	451(42.9)	385(54.2)
K. pnenmoniae	612(31.6)	52(32.3)	352(33.5)	197(27.7)
E. cloacae	58(3.0)	5(3.1)	32(3.0)	21(3.0)
other Enterobacter sp.	55(2.8)	8(5.0)	31(2.9)	16(2.3)
Serratia sp.	28(1.4)	2(1.2)	19(1.8)	7(1.0)
other Klebsiella sp.	25(1.3)	4(2.5)	12(1.1)	9(1.3)
Proteus sp.	13(0.7)	1(0.6)	7(0.7)	5(0.7)
Citrobacter sp.	18(1.0)	2(1.2)	11(1.0)	5(0.7)
Salmonella sp.	8(0.4)	2(1.2)	5(0.5)	1(0.1)
M. morganii	6(0.3)	-	3(0.3)	3(0.4)
Raoultella sp.	4(0.2)	1(0.6)	2(0.2)	1(0.1)
Pantoea sp.	1(0.1)	-	-	1(0.1)
**Vibrionaceae**				
Aeromonas sp.	3(0.2)	-	3(0.3)	-
P. shigelloides	1(0.1)	-	1(0.1)	-
**Non-fermenting gram-negative bacteria**				
P. aeruginosa	95(4.9)	16(9.9)	50(4.8)	28(3.9)
A. baumannii	82(4.2)	6(3.7)	53(8.0)	23(3.2)
other Acinetobacter sp.	11(0.6)	1(0.6)	8(0.8)	2(0.3)
S. maltophilia	11(0.6)	2(1.2)	5(0.5)	4(0.6)
Burkholderia sp	5(0.3)	1(0.6)	3(0.3)	1(0.1)
R. mannitolilytica	2(0.1)	-	1(0.1)	1(0.1)
C. gleum	1(0.1)	-	1(0.1)	-
**Other**				
H. influenzae	4(0.2)	3(1.9)	1(0.1)	-

* There were seventeen strains without age information of the corresponding host including five *E. coli*, eleven *K. pnenmoniae* and one *E. cloacae*.

Supplementary Table 2. Difference in clinical background of different age groups.

	Age (years) (%)			
	≤18	19~65	> 65	*p*-value
primary infection from the community but not the hospitals	38.6	49.8	58.7	0.000*
bloodstream infection from the community but not the hospitals	35.4	45.8	54.8	0.000*
ICU admission	28.0	26.3	26.5	0.903
Death	7.0	5.3	10.6	0.000*

*p < 0.05

Supplementary Table 3. The antimicrobial susceptibilities of 896 *Escherichia coli* strains isolated from bloodstream infections (BSI) in China.

Antibiotic	Breakpoints	Reference	%R	%I	%S	MIC50	MIC90	Geom.Mean	MIC Range
Polymyxin B	S<=2 R>=4	EUCAST	1.0	0.0	99.0	0.25	0.5	0.3	0.25 - 128
Colistin	S<=2 R>=4	EUCAST	1.5	0.0	98.5	0.25	0.5	0.3	0.25 - 128
Ceftazidime/avibactam	S<=8 R>=16	CLSI	0.2	0.0	99.8	0.064	0.064	0.1	0.06 - 256
Aztreonam/avibactam*[*]	S<=4 R>=16	-	0.3	0.0	99.7	0.064	0.064	0.1	0.06 - 64
Amikacin	S<=16 R>=64	CLSI	1.9	0.1	98.0	2	4	1.6	0.25 - 128
Meropenem	S<=1 R>=4	CLSI	2.9	0.2	96.9	0.064	0.064	0.1	0.06 - 256
Imipenem	S<=1 R>=4	CLSI	3.1	0.3	96.5	0.25	0.5	0.2	0.06 - 256
Piperacillin/tazobactam	S<=8 R>=32	CLSI	4.7	0.6	94.8	8	8	9.3	8 - 512
Ertapenem	S<=0.5 R>=2	CLSI	6.1	1.1	92.7	0.064	0.25	0.1	0.06 - 256
Cefoxitin	S<=8 R>=32	CLSI	9.7	3.7	86.6	2	16	3.8	2 - 128
Cefoperazone/sulbactam	S<=16 R>=64	CLSI	8.0	6.9	85.0	8	32	11.9	8 - 256
Ceftazidime	S<=4 R>=16	CLSI	23.2	7.8	69.0	1	64	1.6	0.06 - 256
Cefepime	S<=2 R>=16	CLSI	32.5	14.1	53.5	2	128	1.6	0.06 - 256
Sitafloxacin[*]	S<=0.25 R>=0.5	EUCAST	50.9	0.0	49.1	0.5	2	0.2	0.01 - 64
Levofloxacin	S<=0.5 R>=2	CLSI	52.1	5.2	42.6	2	16	1.5	0.01 - 64
Ceftriaxone	S<=1 R>=4	CLSI	57.8	0.0	42.2	64	256	6.3	0.06 - 256
Trimethoprim/sulfamethoxazole	S<=2 R>=4	CLSI	65.3	0.0	34.7	8	8	4.7	2 - 8

[*] The breakpoint of Aztreonam/avibactam* and Sitafloxacin refers to the breakpoint of aztreonam and moxifloxacin, respectively.

Supplementary Table 4. The antimicrobial susceptibilities of 612 *Klebsiella pneumoniae* strains isolated from bloodstream infections (BSI) in China.

Antibiotic	Breakpoints	Reference	%R	%I	%S	MIC50	MIC90	Geom.Mean	MIC Range
Polymyxin B	S<=2 R>=4	EUCAST	2.9	0.0	97.1	0.5	0.5	0.5	0.25 - 128
Colistin	S<=2 R>=4	EUCAST	2.9	0.0	97.1	0.25	0.5	0.4	0.25 - 128
Aztreonam/avibactam*[*]	S<=4 R>=8	-	0.2	0.0	99.8	0.064	0.064	0.1	0.06 - 16
Ceftazidime/avibactam	S<=8 R>=16	CLSI	0.5	0.0	99.5	0.064	0.064	0.1	0.06 - 256
Amikacin	S<=16 R>=64	CLSI	21.6	0.2	78.3	1	128	3	0.25 - 128
Meropenem	S<=1 R>=4	CLSI	25.0	0.2	74.8	0.064	256	0.5	0.06 - 256
Imipenem	S<=1 R>=4	CLSI	25.2	1.1	73.7	0.25	128	1.2	0.06 - 256
Ertapenem	S<=0.5 R>=2	CLSI	27.3	1.3	71.4	0.064	256	0.6	0.06 - 256
Piperacillin/tazobactam	S<=8 R>=32	CLSI	26.3	2.3	71.4	8	> 256	23.9	8 - 512
Cefoperazone/sulbactam	S<=16 R>=64	CLSI	28.8	4.1	67.2	8	256	22.7	8 - 256
Cefoxitin	S<=8 R>=32	CLSI	32.2	3.1	64.7	2	128	8.7	2 - 128
Sitafloxacin[*]	S<=0.25 R>=0.5	EUCAST	36.3	0.0	63.7	0.125	16	0.2	0.01 - 64
Ceftazidime	S<=4 R>=16	CLSI	33.8	3.1	63.1	0.5	256	2.1	0.06 - 256
Levofloxacin	S<=0.5 R>=2	CLSI	37.3	4.1	58.7	0.5	64	0.8	0.01 - 64
Cefepime	S<=2 R>=16	CLSI	39.1	3.9	57.0	0.125	256	1.9	0.06 - 256
Ceftriaxone	S<=1 R>=4	CLSI	46.7	0.3	52.9	0.25	256	3	0.06 - 256
Trimethoprim/sulfamethoxazole	S<=2 R>=4	CLSI	48.0	0.0	52.0	2	8	3.7	2 - 8

[*] The breakpoint of Aztreonam/avibactam* and Sitafloxacin refers to the breakpoint of aztreonam and moxifloxacin, respectively.

Supplementary Table 5. The antimicrobial susceptibilities of 95 *Pseudomonas aeruginosa* strains isolated from bloodstream infections (BSI) in China.

Antibiotic	Breakpoints	Reference	%R	%I	%S	MIC50	MIC90	Geom.Mean	MIC Range
Polymyxin B	S<=4 R>=8	EUCAST	1.1	0.0	98.9	1	1	0.7	0.25 - 16
Colistin	S<=4 R>=8	EUCAST	1.1	0.0	98.9	1	1	0.9	0.25 - 64
Ceftazidime/avibactam	S<=8 R>=16	CLSI	3.2	0.0	96.8	1	4	1.3	0.06 - 256
Amikacin	S<=16 R>=64	CLSI	5.3	0.0	94.7	2	8	3.1	0.5 - 128
Sitafloxacin*	S<=1 R>=4	EUCAST	5.3	4.2	90.5	0.125	1	0.2	0.01 - 32
Aztreonam/avibactam**	S<=8 R>=32	-	5.3	9.5	85.3	4	16	3.6	0.06 - 32
Cefepime	S<=8 R>=32	CLSI	10.5	8.4	81.1	2	32	3.9	0.5 - 256
Ceftazidime	S<=8 R>=32	CLSI	13.7	5.3	81.1	2	64	4.4	0.5 - 256
Piperacillin/tazobactam	S<=16 R>=128	CLSI	11.6	11.6	76.8	8	128	15.9	8 - 512
Meropenem	S<=2 R>=8	CLSI	21.1	4.2	74.7	0.5	64	1.1	0.06 - 256
Levofloxacin	S<=1 R>=4	CLSI	18.9	7.4	73.7	0.5	8	0.9	0.125 - 64
Imipenem	S<=2 R>=8	CLSI	27.4	5.3	67.4	2	32	3.1	0.125 - 256
Cefoperazone/sulbactam*	-	-	-	-	-	8	64	14.6	8 - 256

* The breakpoint of Aztreonam/avibactam*, sitafloxacin and cefoperazone/sulbactam refers to the breakpoint of aztreonam, levofloxacin and cefoperazone, respectively.

Supplementary Table 6. The antimicrobial susceptibilities of 82 *Acinetobacter baumannii* strains isolated from bloodstream infections (BSI) in China.

Antibiotic	Breakpoints	Reference	%R	%I	%S	MIC50	MIC90	Geom.Mean	MIC Range
Polymyxin B	S<=2 R>=4	EUCAST	3.7	0.0	96.3	0.5	1	0.5	0.25 - 128
Colistin	S<=2 R>=4	EUCAST	3.7	0.0	96.3	0.5	1	0.7	0.25 - 128
Sitafloxacin*	S<=2 R>=8	EUCAST	4.9	9.8	85.4	1	4	0.7	0.01 - 16
Ceftazidime/avibactam*	S<=8 R>=32	-	0.0	15.9	84.1	4	16	4.4	0.06 - 16
Amikacin	S<=16 R>=64	CLSI	64.6	0.0	35.4	128	128	29.4	0.5 - 128
Trimethoprim/sulfamethoxazole	S<=2 R>=4	CLSI	72.0	0.0	28.0	8	8	5.1	2 - 8
Imipenem	S<=2 R>=8	CLSI	70.7	1.2	28.0	64	128	15.9	0.125 - 256
Ceftazidime	S<=8 R>=32	CLSI	74.4	0.0	25.6	64	256	51.4	1 - 256
Meropenem	S<=2 R>=8	CLSI	74.4	1.2	24.4	64	128	17.9	0.125 - 256
Piperacillin/tazobactam	S<=16 R>=128	CLSI	74.4	2.4	23.2	256	> 256	114.7	8 - 512
Levofloxacin	S<=2 R>=8	CLSI	61.0	15.9	23.2	8	32	3.9	0.032 - 64
Cefepime	S<=8 R>=32	CLSI	75.6	4.9	19.5	64	256	40.9	0.5 - 256
Ceftriaxone	S<=8 R>=64	CLSI	78.0	8.5	13.4	256	256	113.7	0.06 - 256
Ertapenem	S<=0.5 R>=1	CLSI	98.8	0.0	1.2	256	256	75.2	0.5 - 256
Aztreonam/avibactam*	-	-	-	-	-	16	64	23.6	0.06 - 128
Cefoperazone/sulbactam	-	-	-	-	-	32	128	36.6	8 - 256
Cefoxitin	-	-	-	-	-	128	128	104.5	16 - 128

[*] The breakpoint of Ceftazidime/avibactam and sitafloxacin refers to the breakpoint of ceftazidime and levofloxacin, respectively.

Supplementary Table 7. The antimicrobial susceptibilities of 58 *Enterobacter cloacae* strains isolated from bloodstream infections (BSI) in China.

Antibiotic	Breakpoints	Reference	%R	%I	%S	MIC50	MIC90	Geom.Mean	MIC Range
Polymyxin B	S<=2 R>=4	EUCAST	19.0	0.0	81.0	0.5	128	1.1	0.25 - 128
Colistin	S<=2 R>=4	EUCAST	20.7	0.0	79.3	0.25	128	1.1	0.25 - 128
Ceftazidime/avibactam	S<=8 R>=16	CLSI	0.0	0.0	100.0	0.064	0.064	0.1	0.06 - 4
Aztreonam/avibactam*[*]	S<=4 R>=8	-	1.7	0.0	98.3	0.064	0.125	0.1	0.06 - 32
Amikacin	S<=16 R>=64	CLSI	3.4	0.0	96.6	1	4	1.6	0.5 - 128
Meropenem	S<=1 R>=4	CLSI	8.6	0.0	91.4	0.064	0.25	0.1	0.06 - 256
Ertapenem	S<=0.5 R>=2	CLSI	10.3	5.2	84.5	0.125	2	0.2	0.06 - 256
Piperacillin/tazobactam	S<=8 R>=32	CLSI	10.3	6.9	82.8	8	32	11.3	8 - 512
Cefoperazone/sulbactam	S<=16 R>=64	CLSI	6.9	10.3	82.8	8	32	12.3	8 - 256
Imipenem	S<=1 R>=4	CLSI	13.8	6.9	79.3	0.5	4	0.7	0.06 - 128
Sitafloxacin[*]	S<=0.25 R>=0.5	EUCAST	22.4	0.0	77.6	0.012	2	0.1	0.01 - 8
Cefepime	S<=2 R>=16	CLSI	15.5	6.9	77.6	0.064	128	0.5	0.06 - 256
Levofloxacin	S<=0.5 R>=2	CLSI	19.0	8.6	72.4	0.064	8	0.2	0.01 - 32
Ceftazidime	S<=4 R>=16	CLSI	32.8	1.7	65.5	0.5	128	2.1	0.125 - 256
Ceftriaxone	S<=1 R>=4	CLSI	43.1	5.2	51.7	1	256	2.8	0.06 - 256
Trimethoprim/sulfamethoxazole	S<=2 R>=4	CLSI	51.7	0.0	48.3	4	8	3.9	2 - 8
Cefoxitin	S<=8 R>=32	CLSI	87.9	1.7	10.3	128	128	73	2 - 128

[*] The breakpoint of Aztreonam/avibactam* and sitafloxacin refers to the breakpoint of aztreonam and moxifloxacin, respectively.

Supplementary Table 8. Antimicrobial susceptibility rates of Gram-negative bacteria isolates to polymyxin B and colistin in different clinical features.

Clinical features	Polymyxin B				Colistin			
	Yes	No	*P*-value	OR	Yes	No	*P*-value	OR
Pulmonary diseases	93.7	94.2	0.654	0.909	93.7	93.4	0.81	1.051
Tumors	94.6	93.9	0.566	1.134	94.0	93.2	0.527	1.141
Hepatobiliary diseases	94.5	93.8	0.556	1.129	94.2	93.0	0.327	1.214
Digestive system diseases	94.2	94.0	0.884	1.039	93.6	93.4	0.881	1.038
Cardiovascular system diseases	93.7	94.2	0.710	0.922	93.5	93.4	0.937	1.017
Nervous system diseases	93.1	94.3	0.455	0.816	93.1	93.6	0.770	0.924
Urinary system diseases	93.9	94.1	0.822	0.951	93.9	93.3	0.682	1.094
Diabetes	95.2	93.7	0.245	1.326	94.5	93.1	0.283	1.279
Drinking history	96.6	93.7	0.075	1.920	96.6	93.0	0.035*	2.152
Smoking history	95.3	93.9	0.362	1.316	95.3	93.1	0.187	1.482
Agranulosis	96.0	93.9	0.243	1.586	94.9	93.3	0.409	1.34
Hypoproteinemia	95.2	93.1	0.045*	1.481	94.4	92.6	0.096	1.364
Splenectomy history	84.6	94.1	0.178	0.343	84.6	93.5	0.208	0.382
Operation history (in recent three months)	94.3	94.0	0.956	~1.000	93.3	94.3	0.786	~0.800
Hormone usage (in recent three months)	94.6	94.0	0.725	1.117	94.6	93.3	0.464	1.256
Immunosuppressor usage (in recent three months)	94.4	94.0	0.848	1.071	93.2	93.5	0.880	0.952
Medical institution admission (in recent three months)	94.1	94.0	0.967	~1.000	93.3	93.7	0.909	~1.000
Antibiotics usage (in recent three months)	95.8	93.4	0.045	1.609	95.6	92.6	0.016	1.738
Indwelling catheter usage (in recent three months)	94.6	93.9	0.614	1.131	94.6	93.1	0.294	1.288
Fever exceeds > 39℃	93.9	94.2	0.763	0.943	93.1	93.8	0.561	0.898

*$p < 0.05$

Supplementary Table 9. Susceptibility of Gram-negative bacteria of different infection sources to polymyxin B and colistin.

N (percentage)	polymyxin B		Total	colistin		Total
	R	S		R	S	
Respiratory tract infection	28(8.2)	313(91.7)	341	32(9.3)	309(90.6)	341
Urinary tract infection	9(3.4)	254(96.5)	263	9(3.4)	254(96.5)	263
Alimentary tract Infection	0(0.0)	51(100.0)	51	1(1.9)	50(98.0)	51
Central nervous system infection	1(6.2)	15(93.7)	16	2(12.5)	14(87.5)	16
Liver abscess	2(2.4)	80(97.5)	82	2(2.4)	80(97.5)	82
Biliary tract infection	10(5.0)	187(94.9)	197	11(5.5)	186(94.4)	197
Abdominal infection of other organs (except liver and biliary tract infection)	14(8.6)	147(91.3)	161	16(9.9)	145(90.0)	161
Pelvic infection (including puerperal infection)	1(5.8)	16(94.1)	17	1(5.8)	16(94.1)	17
Skin and soft tissue infection	1(1.4)	69(98.5)	70	2(2.8)	68(97.1)	70
Cardiovascular system infection	0(0.0)	5(100.0)	5	0(0.0)	5(100.0)	5
Catheter-related bloodstream infections	4(9.5)	38(90.4)	42	4(9.5)	38(90.4)	42
Others	45(6.4)	649(93.5)	694	47(6.7)	647(93.2)	694
**Total**	**115(5.9)**	**1824(94.0)**	**1939**	**127(6.5)**	**1812(93.4)**	**1939**

Supplementary Table 10. Susceptibility Changes of Gram-negative bacteria in bloodstream infection to polymyxin B or colistin in Different Regions of China between 2019-2021.

N (percentage)		Polymyxin B		Colistin	
	N	R(%)	S(%)	R(%)	S(%)
North	363	23(6.3)	340(93.7)	23(6.3)	340(93.7)
East	365	34(9.3)	331(90.7)	37(10.1)	328(89.9)
Northeast	200	13(6.5)	187(93.5)	17(8.5)	183(91.5)
Central	375	18(4.8)	357(95.2)	18(4.8)	357(95.2)
South	196	6(3.1)	190(96.9)	6(3.1)	190(96.9)
Southwest	309	13(4.2)	296(95.8)	17(5.5)	292(94.5)
Northwest	131	8(6.1)	123(93.9)	9(6.9)	122(93.1)
**Total**	**1939**	**115(5.9)**	**1824(94.1)**	**127(6.5)**	**1812(93.5)**

Supplementary Table 11. Susceptibility Changes of 5 main Gram-negative bacteria in bloodstream infection to polymyxin B in Different Regions of China between 2019-2021.

	Escherichia coli			*Klebsiella pneumoniae*			*Pseudomonas aeruginosa*			*Acinetobacter baumannii*			*Enterobacter cloacae*		
	R(%)	S(%)	total	R(%)	S(%)	total	R(%)	S(%)	total	R(%)	S(%)	total	R(%)	S(%)	total
North	4(2.6)	147(97.4)	151	2(1.7)	115(98.3)	117	0(0.0)	20(100.0)	20	1(4.3)	22(95.7)	23	1(12.5)	7(87.5)	8
East	2(1.4)	142(98.6)	144	8(6.8)	110(93.2)	118	1(3.4)	28(96.6)	29	0(0.0)	13(100.0)	13	3(17.6)	14(82.4)	17
Northeast	0(0.0)	77(100.0)	77	1(1.5)	64(98.5)	65	0(0.0)	10(100.0)	10	1(7.7)	12(92.3)	13	0(0.0)	6(100.0)	6
Central	2(1.1)	177(98.9)	179	2(1.6)	121(98.4)	123	0(0.0)	16(100.0)	16	1(6.2)	15(93.8)	16	4(28.6)	10(71.4)	14
South	1(0.9)	115(99.1)	116	0(0.0)	63(100.0)	63	0(0.0)	3(100.0)	3	0(0.0)	7(100.0)	7	2(66.7)	1(33.3)	3
Southwest	0(0.0)	172(100.0)	172	2(2.7)	73(97.3)	75	0(0.0)	12(100.0)	12	0(0.0)	9(100.0)	9	0(0.0)	6(100.0)	6
Northwest	0(0.0)	57(100.0)	57	3(5.9)	48(94.1)	51	0(0.0)	5(100.0)	5	0(0.0)	1(100.0)	1	1(25.0)	3(75.0)	4
**total**	**9(1.0)**	**887(99.0)**	**896**	**18(2.9)**	**594(97.1)**	**612**	**1(3.4)**	**94(98.9)**	**95**	**3(3.7)**	**79(96.3)**	**82**	**11(19.0)**	**47(81.0)**	**58**
